# Production and assay of cellulolytic enzyme activity of *Enterobacter cloacae* WPL 214 isolated from bovine rumen fluid waste of Surabaya abbatoir, Indonesia

**DOI:** 10.14202/vetworld.2015.367-371

**Published:** 2015-03-21

**Authors:** W. P. Lokapirnasari, D. S. Nazar, T. Nurhajati, K. Supranianondo, A. B. Yulianto

**Affiliations:** 1 Department of Animal Husbandry, Faculty of Veterinary Medicine, Airlangga University, Jl. Mulyorejo, Campus C Unair, Surabaya, Indonesia; 2 Faculty of Veterinary Medicine, Wijaya Kusuma Surabaya University, Jl. Dukuh Kupang Barat XVI/1 Surabaya, Indonesia

**Keywords:** *endo-(1,4)-β-D-glucanase*, *exo-(1,4)-β-D-glucanase*, *β-glucosidase*, *Enterobacter cloacae* WPL 214

## Abstract

**Aim:**

This study aims to produce and assay cellulolytic enzyme activity (*endo-(1,4)-β-D-glucanase, exo-(1,4)-β-D-glucanase*, and *β-glucosidase*, at optimum temperature and optimum pH) of *Enterobacter cloacae* WPL 214 isolated from bovine rumen fluid waste of Surabaya Abbatoir, Indonesia.

**Materials and Methods:**

To produce enzyme from a single colony of *E. cloacae* WPL 214, 98 × 10^10^ CFU/ml of isolates was put into 20 ml of liquid medium and incubated in a *shaker incubator* for 16 h at 35°C in accordance with growth time and optimum temperature of E. cloacae WPL 214. Further on, culture was centrifuged at 6000 rpm at 4°C for 15 min. Pellet was discarded while supernatant containing cellulose enzyme activity was withdrawn to assay *endo-(1,4)-β-D-glucanase, exo-(1,4)-β-D-glucanase*, and *β-glucosidase*.

**Results:**

Cellulase enzyme of *E. cloacae* WPL 214 isolates had endoglucanase activity of 0.09 U/ml, exoglucanase of 0.13 U/ml, and cellobiase of 0.10 U/ml at optimum temperature 35°C and optimum pH 5.

**Conclusion:**

*E. cloacae* WPL 214 isolated from bovine rumen fluid waste produced cellulose enzyme with activity as cellulolytic enzyme of *endo-(1,4)-β-D-glucanase, exo-(1,4)-β-D-glucanase* and *β-glucosidase*.

## Introduction

Rumen is an excellent environment for microbial growth consisting of bacteria, fungi and protozoa which are widely known to play important role in the fermentation process of ruminant cattle feed [1]. Cellulase is an enzyme produced by cellulolytic microbes capable of hydrolizing β-1,4 glycoside bond in cellulose, a polysaccharide structure often found in plants [2].

Cellulose degradation by cellulolytic bacteria is a product of synergy in a group of cellulase enzymes. Cellulase enzyme system consists of three groups of hydrolytic enzymes, i.e. (1) *endo-(1,4)-β-D-glucanase* (endoglucanases), (2) *exo-(1,4)-β-D-glucanase (exoglucanases)*, and (3) *β -glucosidase* [3].

*Endo-(1,4)-β-D-glucanase* enzyme hydrolyzes β bonds randomly in a morphous regions of cellulose fibers [4], generates oligosaccharides of different lengths, and can form a new chainend [5]. *Exo-(1,4)-β-D-glucanase* enzyme works towards reducing and non-reducing end of polysaccharide chains, especially on *crystalline cellulose* region, and liberates glucose as the main product resulted by *β-glucosidase* enzyme. Hydrolysis of crystal line cellulose part can only be done efficiently by *exoglucanase* enzyme. The synergy between *endoglucanases* and *exoglucanases* enzymes produces cellobiose molecules. Cellulose hydrolysis effectively requires an enzyme (β-glucosidase) that breaks down cellobiose into two molecules of glucose [5,6].

The cost of using commercial cellulose enzymes is still expensive, making it less economical when applied in livestock industry in relation to provision and improvement of feed quality. Thus, other cellulolytic microbes capable of degrading fibrous feed stuffs need developing. Biodegradation by *Enterobacter cloacae* WPL 214 rumen cellulolytic bacteria is expected to be able to be used as degrading source material for fibrous feed stuffs at a cheaper price compared to commercial cellulose enzymes.

## Materials and Methods

### Materials

#### Cellulolytic enzyme activity

Media used in this study were Luria Bertani medium (1 g trypton, 0.5 g *bacto yeast extract* and 1 g NaCl); dinitrosalicylic acid (C_4_H_4_KNaO_6_.4H_2_O), (Merck); Natriumhydroxide (NaOH), (Merck); 3,5 - dinitrosalicylic acid, (Sigma); Sodium Sulfite (Na_2_SO_3_), citric acid, Na_2_HPO_4_.7H_2_O, NaHPO_4_, p-nitrophenyl cellobioside (PNPC), carboximethyl cellulose (CMC), p-nitrophenyl-β-D-glukopiranoside (pNPG), p-nitrophenol.

### Methods

#### Measurement of E. cloacae WPL 214 growth curve

10 ml of cultured *E. cloacae* WPL 214 isolates was taken and transferred to100 ml growth medium in Erlenmeyer flask. Culture suspensions were incubated in a *shaker incubator* (37°C, 120 rpm). Optical density (OD) was measured at λ 600 nm by taking as much as 1 ml sampling with interval of 2 h for 30 h (h 0; 2; 4; 6; 10; 12; 14; 16; 18; 20; 22; 24; 26; 28; 30). The first sampling was done at 0th h and continued until OD values showed a clear decline. OD was measured with UV-vis spectrophotometer at λ 600 nm. Growth curve was obtained from the result of absorbance measurement on the time.

#### Measurement of E. cloacae WPL 214 optimum temperature and pH

1 ml of cultured cellulolytic bacteria isolates of *E. cloacae* WPL 214 was taken and transferred into 10 ml Luria Bertani growth medium in erlenmeyer flask. Culture suspensions were incubated for 24 h in a shaker incubator at 30°C, 35°C, 40°C and pH 4, 5, 6, 7, 8 with shaking at 120 rpm. OD measurement was conductedby taking 1 ml sampling. OD was measured using UV-Vis spectrophotometer at λ 600 nm.

### Cellulase enzyme production of *E. cloacae* WPL 214

Enzyme activity assay was conducted on enzyme produced from optimum growth time and temperature in the following way: Single colony of *E. cloacae* of 98 × 10^10^ CFU/mlwas put into 20 ml of liquid medium and incubated in a *shaker incubator* for 16 h at 35°C. Afterwards, culturewas centrifuged at 6000 rpm at 4°C for 15 min. Pellet was discarded while supernatant containing cellulase enzyme was taken to assay *endo-(1,4)-β-D-glucanase*, exo-(1,4)-β-D-glucanase, and *β-glucosidase* enzyme activity.

### Assay of *endo-(1,4)-β -D-glucanase E. cloacae* WPL 214 activity

Assay of *endo-(1,4)-β-D-glucanase* activity was conducted using 3.5-dinitrosalisilat acid (DNS) method by using CMC as a specific substrate. *Endo-(1,4)-β-D-glucanase* activity was assayed by mixing 100 ml enzyme with 100 ml substrate(1% CMC in 0.1M citratephosphate bufferat pH7) and incubating it in a water bath at 50°C for 30 min. Enzyme activity of an amount of formed reducing sugars was measured using DNS method by adding 600 µl DNS into tube and placing it in a boiling water bath for 15 min together with control (containing 100 µl enzyme mixed with 600 µl and 100 µl substrate, without incubation) and finally cooling it in ice water for 20 min. Total volume of enzyme activity assay in this study was 800 µl. Absorbance measurement was afterward conducted using cuvettes. Absorbance was read using spectrophotometer at λ 550 nm. One unit of enzyme activity was defined as the amount of enzyme required to form 1 mmol of product per unit time for each ml of enzyme [7,8].

### Assay of *exo-(1,4)-β-D-glucanase E. cloacae* WPL 214 activity

To measure *exo-(1,4)-β-D-glucanase* activity, 100 μl enzyme mixed with 900 μl pNPC (1 mMol *p*NPC in 10 ml of citrate phosphate buffer at pH5) was incubated for 30 min at optimum temperature 35°C. Reaction was stopped by adding 100 μl 1M Na_2_CO_3_. Liberation of p-nitrophenol was read using spectrophotometer at λ 405 nm. A sa blank, 100 μl aquadest and 900 μl substrate that were treated the same as sample condition were used and reaction was stopped by adding 100 μl of 1M Na_2_CO_3_. One unit of enzyme activity is equivalent to the amount of enzyme required to produce 1 μmol p-nitrophenol/min [9].

p-nitrophenol standard was made in the range of 0.1-0.5 mM p-nitrophenol from 10 mM p-nitrophenol stock in phosphate citrate buffer solvent pH 5. 100 μl of each standard p-nitrophenol solution was mixed with 900 μl of phosphate citrate buffer pH 6 and incubated at 35°C for 30 min. Reaction was stopped by adding 100 μl of 1 M Na_2_CO_3_ and absorbance was read using UV-Vis spectrophotometer at λ 405 nm.

### Assay of *β-glucocidase E. cloacae* WPL 214 activity

Measurement of *β-glucocidase* activity was assayed using *p*NPG method. *β-glucocidase* activity was measured by mixing 100 μl enzyme with 900 μl substrate (1 mM *p*NPG in 10 ml of phosphate citrate buffer pH5) and incubating it at 35°C for 30 min. Reaction was stopped by adding 100μl of 1M Na_2_CO_3_. As blank, 100 μl aquadest and 900 μl substrate that were treated the same as sample condition were used. Liberation of p-nitrophenol was read with spectrophotometer at λ 405 nm and then compared top-nitrophenol standard curve. One unit of enzyme activity was equivalent to the amount of enzyme required to produce 1 μmol p-nitrophenol/min [9].

### Statistical analysis

This research was descriptive research so that the duplo data collected and analyzed in descriptive and expressed in the form of narrative and figures.

## Results

### *E. cloacae* WPL 214 growth curve

**Figure-1:**
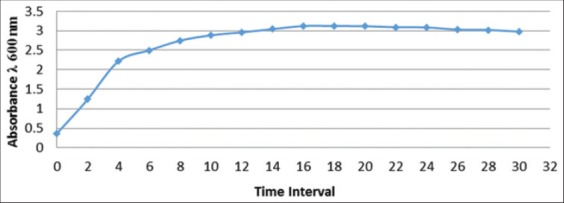
Growth curve of *Enterobacter cloacae* WPL 214 on liquid medium every 2 h for 30 h

Growth curve of *E. cloacae* WPL 214 cellulolytic bacterial inoculants is shown in Figure-1. The highest growth logarithmic phase occurred at the 16^th^ h with absorbance (λ 600 nm) of 3.122.

### Optimum temperature and pH of cellulase enzyme *E. cloacae* WPL 214

Incubation temperature was specified at 30°C, 35°C, 40°C and 45°C and phosphate citrate at pH 4, 5, 6, 7, 8. The highest cellulose enzyme activity was obtained at 35°C as much as 0.154 U/ml (Table-1) and at pH5 as much as 0.606 U/ml (Table-2).

**Table-1:** Mean of activity of cellulase enzyme of *E. cloacae* WPL 214 data (U/ml) at certain temperature.

Temperature (°C)	Activity (U/mL)±SD
30	0.073148±0.000327
35	0.154167±0.000655
40	0.078241±0.000982
45	0.037963±0.000327

*E. cloacae=Enterobacter cloacae*, SD=Standard deviation

**Table-2:** Mean of activity of cellulase enzyme of *E. cloacae* WPL 214 Data (U/ml) at certain pH.

pH	Activity (U/mL)±SD
4	0.435185±0.000655
5	0.606019±0.000327
6	0.430093±0.006547
7	0.321759±0.000982
8	0.220833±0.000655

*E. cloacae=Enterobacter cloacae*, SD=Standard deviation

### Cellulase enzyme production of *E. cloacae* WPL 214

**Figure-2:**
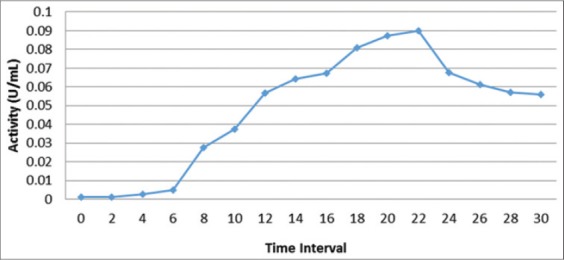
Growth curve of *Enterobacter cloacae* WPL 214 on liquid medium every 2 h for 30 h

In addition to growth curve observation, measurement of cellulase enzyme production was also conducted. Optimum production of cellulase enzyme occurred at the end of stationary phase, at the same time as the peak of microbial growth which occured at the 22^nd^ h (Figure-2).

## Discussion

### *E. cloacae* WPL 214 growth curve

Growth curve describes gradual growth process of a microorganism, from the beginning until the end of activity. This consists of four main phases: Lag, exponential, stationary, and death [10]. During this phase mass or cell accretion has not happened yet. Therefore, phase curve is generally flat. Lag phase interval depends on the compatibility between activity and environment setting. In this research, this phase occurs before in the first 2 h of *E. cloacae* WPL 214 isolate growth and followed by exponential phase.

Exponential or logarithmic phase is a phase when transformation activity increases and the accretion of microorganism growth reaches maximum speed so that the curve is in exponential form. This increasing activity should be offset by many factors among others: Biological factors, such as shape and nature of the microorganism to its environment, life association between related organism, and non-biological factors, such as temperature, pH, and nutrient content in the growth medium. Exponential phase of *E. cloacae* WPL 214 isolates occurred during the first 2 h of incubation up to the 8^th^ h of incubation.

Stationary phase is a phase when increased and decreased activities come to a balance. In colony growth, it means the rate of individual death equals the rate of individual birth. Therefore, this phase forms a flat curve. This phase also occurs due to diminishing nutrient sources, inhibitory compounds formation, and unfavorable environmental factors. Stationary phase of *E. cloacae* WPL 214 isolates occurred from the 8^th^ up to the 28^th^ h of incubation with optimum growth at 16th h of incubation.

Death phase is the phase when cessation of activity starts, or in colony growth, the event of death begins to exceed individual birth. Death phase of *E. cloacae* WPL 214 isolate soccured after 30 h of incubation.

### Cellulase enzyme production of *E. cloacae* WPL 214

In addition to growth curve observation, measurement of cellulase enzyme production was also conducted. Optimum production of cellulase enzyme occurred at the end of stationary phase, at the same time as the peak of microbial growth which occurred at the 22^nd^ h (Figure-2). Cellulolytic activity of cellulose enzyme was assayed by performing reaction between cellulase enzyme and p-nitrophenol substrate derivatives. Exoglucanase was assayed using *p*NPC specific substrate while cellobiase was assayed using *p-nitrophenyl-β-D-glucopyranocide* (*p*NPG) specific substrate. Enzyme activity was determined by measuring the amount of p-nitrophenol released [11]. Observations on the amount of p-nitrophenol released was observed by using spectrophotometry at λ 405 nm. One unit of cellulase enzyme activity is defined as the amount of enzyme producing 1 μmol of *p*-nitrophenol in 1 min (U/ml) in experimental conditions. Cellulase enzyme activity curve was used to determine the optimum time required to obtain maximum cellulase enzyme activity from enzyme production process.

### Activity of cellulolytic enzyme (*endo-(1,4)-*β-D-glucanase, exoglucanase, and *cellobiase*) of *E. cloacae* WPL 214

Measurement of exoglucanase and β-glucocidase enzyme activity was conducted using p-nitrophenol derivative substrates. Exoglucanase was assayed using *p*NPC specific substrate while cellobiase was assayed using *p*NPG specific substrate. Assays were carried out using the enzyme produced when enzyme activity reached optimum time, at the 22^nd^ h. Assay was also carried out at optimum temperature of 35°C and optimum pH of 5. Data of exoglucanase and cellobiase enzyme activity are presented in Table-3. A unit of enzyme activity is defined as the amount of enzyme required to form 1 μmol product per minute.

**Table-3:** Activity of *E. cloacae* WPL 214 enzyme.

Enzyme	Specific substrates	Activity (U/ml)±SD
*endo-(1,4)-β-D-glucanase*	DNS	0.09±0.00027
*exo-(1,4)-β-D-glucanase*	*p*NPC	0.13±0.0131
*β-glucocidase*	pNPG	0.10±0.0069

pNPC=p- nitrophenyl cellobioside, DNS=3,5-dinitrosalicylic acid, pNPG=p- nitrophenyl -β-D-glucopyranocide, *E. cloacae=Enterobacter cloacae*, SD=Standard deviation

*Endo-(1,4)-β-D-glucanase* enzyme activity was measured simultaneously with the making of the growth curve. Sample was taken every 2 h and the enzyme extracted. Cellulase enzyme activity curve was used to determine the optimum time required to obtain maximum cellulase enzyme activity from enzyme production process. Figure-2 shows data of optimum time needed to obtain optimum growth curve and optimum enzyme activity, which occurred at the 22nd h with enzyme activity of 0.09 U/ml.

Research data of *E. cloacae* WPL 214 isolates showed that the optimum time to produce cellulose enzyme was at the 22^nd^ h with endoglucanase enzyme activity of 0.09 units/ml (U/ml). Results of enzyme activity measurement using specific substrates of p-nitrophenol derivatives on *E. cloacae* WPL 214 isolates showed exoglucanase activity of 0.13 U/ml while cellobiose enzyme activity was at 0.10 U/ml. This result indicated that these isolates had activity on endoglucanase, exoglucanase and cellobiose enzymes. Cellulose hydrolysis requires synergistic activity of various cellulase enzymes with different specifications to produce multi enzyme system. It also requires coordination in cellulose molecule breaking, product resulted, and catalytic movement of cellulose chain [12,13]. Multi enzyme system of cellulose is a strategy of microorganisms to improve the effectiveness of cellulase hydrolysis, in which each enzyme has a specific function [14].

Not all *Enterobacter* bacteria produce cellulase enzyme. Isolation and identification have also been made from several plants, i.e. 53 endophyticentero bacteria from some plants including citrus, coconut, eucalyptus, sugarcane, and soybean. These studies identified *E. cloacae*, *Pantoea agglomerans*, *Hafniaalvei*, and *Pantoea ananatis*. The lowest cellulase enzyme production is obtained from coconut plants as much as 20% (2 of 10), equivalent to that produced by citrus crop as much as 20% (4 of 20). The level of cellulase production obtained, from the highest to the lowest, is as follows: Sugarcane, 100% (4 of 4), eucalyptus, 84.6% (11 of 13), and soybean, 83% (5 of 6) [15]. Isolation and identification of cellulolytic bacteria from insects show the existence of cellulolytic bacteria: *Enterobacter chrysanthemi*, *E. cloacae*, and *Proteus mirabilis*, *Erwinia chrysanthemi*, among others. These microorganisms are already known to have cellulolytic activity. Cellulolytic bacteria found in the digestive tract of insects have higher cellulolytic activity ability as they are naturally involved in the digestion of lignocellulosic substrates found in insect feed. Genetic engineering (recombinant) is performed on those cellulolytic bacteria and is further used in its role of converting cellulosic biomass (CMC substrate and sugarcane *bagasse*) into bioethanol product more quickly and efficiently through process of fermentation at 37°C and pH 7. Cellulosic ethanol products from cellulolytic bacteria aim to reduce dependence on petroleum [16].

*E. cloacae* isolated from pumpkin (*Aulacophora atripenis*) bees can grow on a medium with different carbon sources, including CMC and 2% Avicel to produce *Endo-1,4-β-D-glucanase* enzyme. The maximum production of cellulase enzyme is obtained after 96 h of fermentation. The highest endoglucanase enzyme production occurs when grown in medium of 0.75% CMC. Enzymes *Endo-1,4-β-D-glucanase* is optimum at pH 5.8 and 40°C. The maximum number of enzymes produced on CMC substrates and Avicel 2% is lower, at 0.05 µM/ml, compared to enzyme produced on substrates of 0.75% CMC, 0.9 U/ml [17]. *E. cloacae* WPL 214 isolate in this study also had 0.09 U/ml endoglucanase enzyme activity obtained at optimum time: 22nd h at pH 5 and temperature 35°C.

## Conclusion

Based on the research results, it can be concluded that cellulolytic enzyme having activity of *endo-(1,4)-β-D-glucanase*, *exo-(1,4)-β-D-glucanase* and *β-glucosidase* can be produced from cellulolytic isolates of *E. cloacae* WPL 214 isolated from bovine rumen fluid waste of Surabaya Abbatoir, Indonesia. This indicates that *E. cloacae* WPL 214 can be used to hydrolyze fibrous feeding material containing lignocellulose.

## Authors’ Contributions

WPL designed the research, collected and processed samples. DSN helped in designing the research. ABY carried out the data collection and gathering assay samples. TN and KS assisted in manuscript preparation; WPL, DSN, TN, and KS collected materials for manuscript. All authors have read and approved the final manuscript.
